# Dysregulated miRNAs and their pathogenic implications for the neurometabolic disease propionic acidemia

**DOI:** 10.1038/s41598-017-06420-8

**Published:** 2017-07-18

**Authors:** Ana Rivera-Barahona, Alejandro Fulgencio-Covián, Celia Pérez-Cerdá, Ricardo Ramos, Michael A. Barry, Magdalena Ugarte, Belén Pérez, Eva Richard, Lourdes R Desviat

**Affiliations:** 1 Centro de Biología Molecular Severo Ochoa UAM-CSIC, Universidad Autónoma, Madrid, Spain; 2 Centro de Diagnóstico de Enfermedades Moleculares (CEDEM), Madrid, Spain; 3 Centro de Investigación Biomédica en Red de Enfermedades Raras (CIBERER), ISCIII, Madrid, Spain; 4 Instituto de Investigación Sanitaria Hospital La Paz (IdiPaz), ISCIII, Madrid, Spain; 5 Genomic Facility, Parque Científico de Madrid, Madrid, Spain; 6 Mayo Clinic, Rochester, Minnesota USA

## Abstract

miRNome expression profiling was performed in a mouse model of propionic acidemia (PA) and in patients’ plasma samples to investigate the role of miRNAs in the pathophysiology of the disease and to identify novel biomarkers and therapeutic targets. PA is a potentially lethal neurometabolic disease with patients developing neurological deficits and cardiomyopathy in the long-term, among other complications. In the PA mouse liver we identified 14 significantly dysregulated miRNAs. Three selected miRNAs, miR-34a-5p, miR-338-3p and miR-350, were found upregulated in brain and heart tissues. Predicted targets involved in apoptosis, stress-signaling and mitochondrial function, were inversely found down-regulated. Functional analysis with miRNA mimics in cellular models confirmed these findings. miRNA profiling in plasma samples from neonatal PA patients and age-matched control individuals identified a set of differentially expressed miRNAs, several were coincident with those identified in the PA mouse, among them miR-34a-5p and miR-338-3p. These two miRNAs were also found dysregulated in childhood and adult PA patients’ cohorts. Taken together, the results reveal miRNA signatures in PA useful to identify potential biomarkers, to refine the understanding of the molecular mechanisms of this rare disease and, eventually, to improve the management of patients.

## Introduction

microRNAs (miRNAs) are essential players in gene expression regulation. They are non-coding single-stranded RNAs of 20–24 nucleotides in length that act post-transcriptionally by base-pairing with the 3′ untranslated regions of target mRNAs. Typically, an 8-mer “seed” sequence located in the 5′ end of miRNAs directs the recognition of target mRNA and, consequently, gene silencing by degradation or translational repression, depending on whether the complementarity between miRNA and target mRNA sequence is perfect or not^1, 2^. In some cases alternative modes of miRNA target recognition have been described, including G-bulge sites^3^, imperfect centered sites^4^ or sites centering on miRNA nucleotides 13-16 that compensate for seed mismatches or that supplement the seed region^5^. A single miRNA may control the expression of multiple targets and a particular mRNA can be targeted by several miRNAs, thus establishing miRNAs networks that govern many biological processes including cell differentiation, proliferation, cell death and metabolic control. Thereby, miRNA dysregulation may have a broad impact on cellular physiology contributing to disease development. In fact, alterations in miRNA function have been reported in many human disorders such as cancer^6^, cardiovascular^7^ and neurodegenerative diseases^8, 9^. One of the most exciting developments in the field of miRNA research involves the efficient manipulation of miRNA function using antisense oligonucleotides acting as miRNA inhibitors or antagonists (antagomirs) or synthetic miRNAs (miRNA mimics) for restoring normal levels of a miRNA associated to a disease state. To date, there is a large interest in the potential of this approach which has already entered the clinical phase^10^.

In the past few years, the discovery of the stable presence of miRNAs in body fluids in relation to disease has opened new clinical avenues for miRNAs as diagnostic tools^11^. Circulating miRNAs can originate from damaged cells due to passive leakage or can be actively secreted packaged in exosomes or microvesicles, or conjugated with proteins. Subsequently, they can be taken up by new cells where they can potentially regulate gene expression thus acting as extracellular messengers^12–16^. In clinical settings, the use of circulating miRNAs as minimal invasive biomarkers for diagnosis, prognosis or treatment monitoring has been explored mainly for cancer and cardiovascular diseases^17^.

Recent evidence shows that miRNAs play a role in mitochondrial dysfunction, apoptosis and oxidative stress^18, 19^, which contribute to the pathogenesis of many human disorders, including inherited metabolic diseases (IMD)^20–22^. All IMD are classified as rare diseases due to their low individual prevalence and most of them do not currently have an effective treatment. Among them, propionic acidemia (PA, MIM#606054) is one of the most frequent, life-threatening organic acidemias, with an incidence of 1 in 100,000 live births, and caused by mutations in either the *PCCA* or *PCCB* genes, encoding both subunits of the mitochondrial propionyl-CoA carboxylase (PCC, E.C.6.4.1.3) enzyme. PCC catalyzes the carboxylation of propionyl-CoA, derived from the catabolism of several amino acids, cholesterol side chain and odd-chain fatty acids, to D-methylmalonyl-CoA, which eventually enters the Krebs cycle^23^. *In vivo* and *in vitro* evidence points to the pathogenic role of a secondary mitochondrial dysfunction induced by accumulated toxic metabolites resulting in cellular oxidative damage^24, 25^.

Clinical picture in PA varies from a neonatal toxic encephalopathy to milder later forms with diverse neurological or cardiac symptoms with or without episodes of metabolic decompensation. Advances in supportive treatment based on dietary restriction and carnitine supplementation have allowed patients to live beyond the neonatal period. However, natural progression of PA leads to a multisystemic disorder of gastrointestinal, immune, nervous and cardiovascular system^26, 27^. To date, there is an unmet clinical need to develop novel therapeutic approaches. At present, there is limited understanding of the mechanisms by which PCC deficiency produces multiorgan complications, including the most frequent hypertrophic cardiomyopathy and basal ganglia deterioration, which are primarily responsible for the high morbidity and mortality.

In this study we have investigated the miRNA expression profile in tissue samples from the hypomorphic mouse model of PA *Pcca* ^−/−^(A138T)^28^ and the correlation with specific target genes’ expression. In parallel, we have investigated the circulating miRNA profile in PA patients’ plasma samples. The results provide fundamental new insights into miRNA-mediated altered cellular processes potentially contributing to PA pathogenesis and warrant further studies of circulating miRNAs as PA disease biomarkers.

## Results

### Altered miRNA expression in liver of PA mice

**Figure 1:**
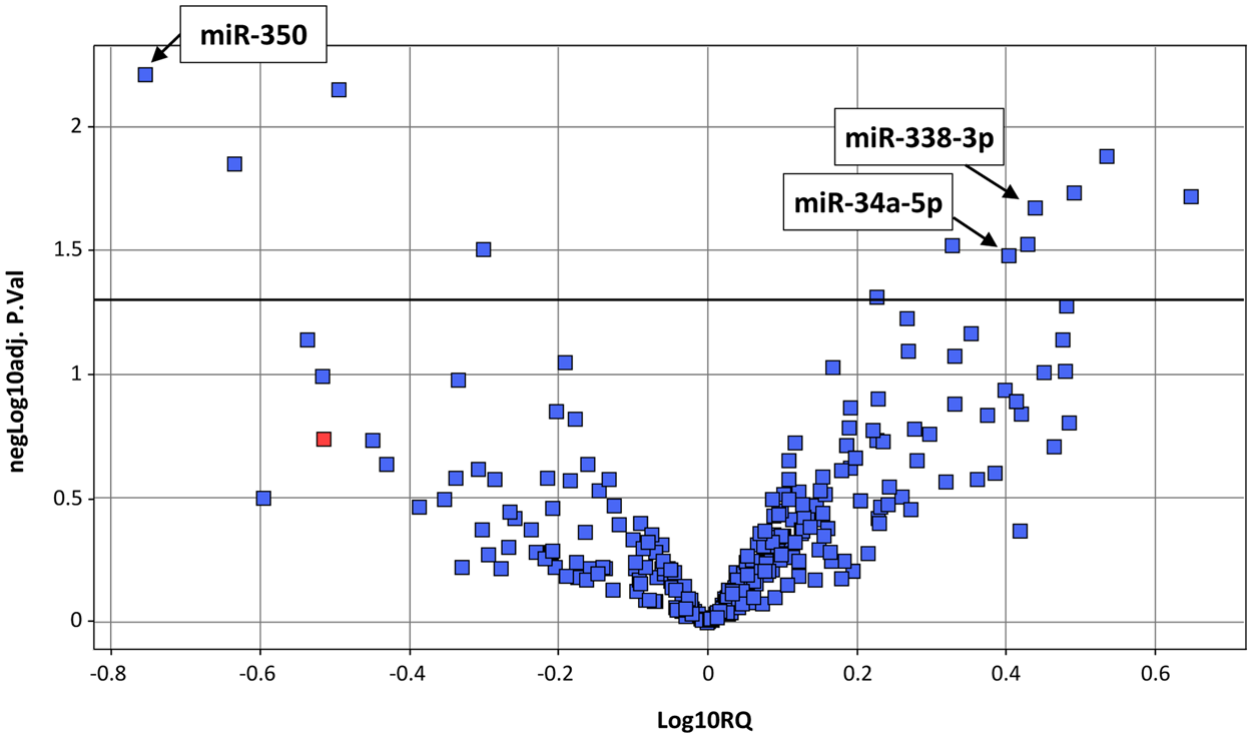
Volcano plot showing the results of the miRNA profile analysis in liver samples of wt and PA mouse. Following qRT-PCR, Ct values were assigned using the SDS2.4 software (Applied Biosystems, Thermo-Fischer) and Ct values above 37,0 were considered as non-detected. MicroRNAs showing very low responses (Ct > 35) in most of the samples of both groups were also removed for further analysis. Finally, Ct values were analyzed using the StatMiner software (Integromics, Perkin-Elmer). The integrated GeNorm software was used to check the stability of putative endogenous genes and a final number of seven was used for normalization (see materials & methods section). Relative comparison of gene expression was made using the wt samples as the reference for the *Pcca* ^−/−^(A138T) group and the geometric mean of selected endogenous genes for normalization. Individual RQ (relative quantity) values as well as statistical *p* values were calculated for the detected 286 miRNAs and represented in log10 scale in the form of a volcano plot.

As a starting point, we performed miRNA profiling in liver tissue of *Pcca* ^−/−^(A138T) (PA) mice in which we had previously detected an altered bioenergetic and redox profile potentially contributing to the pathophysiology of the disease^25^. The analysis was performed by qRT-PCR with PA and wild-type (wt) mice samples (2 months-old, n = 4 per group) using PCR panels with pre-aliquoted primers for 752 miRNAs. We detected 286 target miRNAs with Ct values <35, with 14 miRNAs showing significant differential expression (Fig. 1). Four miRNAs were significantly downregulated (RQ 0.17–0.5) and 10 were upregulated (RQ 1.7–4.5) (Table 1).

**Table 1:** Dysregulated miRNAs in PA mouse liver.

miRNA	RQ	Validated target genes	Biological process	Role in disease	References
miR-31-3p	4.5	*RhoA*	Proliferation and migration	Cancer	53
miR-691	3.4	No data	No data	No data	
miR-700-3p	3.1	No data	No data	No data	
miR-29a-5p	3.1	No data	No data	Cancer	54
miR-501-3p	3	*Gria1*	Neuro-transmision	No data	55
miR-338-3p*	2.7	*Aatk, Atp5g1, CoxIV*	Axonal guidance, apoptosis, mitochondrial function	Cancer, neurodegeneration	42, 56
miR-139-3p	2.7	*MMP11*	Extracellular matrix organization	Cancer	57
miR-34a-5p*	2.5	*Bcl-2, Notch1, Map2k1, Sirt1*	Apoptosis, mitochondrial function, oxidative stress response	Cancer, Alzheimer, cardiomyopathy	29, 30, 34, 43, 44
miR-335-3p	2.1	Ank3	No data	No data	58
miR-1949	1.7	*Rb1*	Cell cycle control	Cancer	59
miR-326-3p	0.5	*Bcl-xl, Notch1/2*	Apoptosis, proliferation	Cancer	60, 61
miR-671-5p	0.3	*Smarcb1*	Proliferation	Cancer	62
miR-503-3p	0.2	No data	No data	No data	
miR-350*	0.2	*p38, Jnk*	Apoptosis	Cardiac hypertrophy	32

^*^miRNAs selected for further studies.

Ingenuity Pathway Analysis (IPA) of the miRNA profiling data showed several overrepresented biological functions including cell-to-cell signaling and interaction, cellular proliferation and cellular development. The top enriched functions in pathological processes include cardiac hypertrophy, liver damage and renal necrosis. We performed *in silico* analysis of predicted and validated targets of the dysregulated miRNAs using different bioinformatic tools and databases (miRBase TargetScan, MiRTarBase), as well as a review of published studies including analyzed targets and pathways involved in disease processes for each miRNA. We and other authors have reported that mitochondrial dysfunction and oxidative stress are probable key players in the pathophysiology of PA^24, 25^, which led us to select three miRNAs for further studies, miR-34a-5p, miR-338-3p and miR-350, due to their involvement in these processes. miR-34a-5p targets *Bcl2*, *Sirt1* and *Notch1* genes, influencing apoptosis and mitochondrial energy metabolism, among other processes^29, 30^; miR-338-3p regulates the expression of several subunits of mitochondrial oxidative phosphorylation complexes^31^, and miR-350 regulates p38 and JNK stress kinases^32^.

### miR-34a-5p, miR-338-3p and miR-350 are up-regulated in different tissues of PA mice

Natural history of PA indicates that neurological and cardiac complications are frequent^26, 27^. The hypomorphic PA mouse model used in this study accurately recapitulates biochemical and clinical biomarkers similar to those in patients with PA. Elevated brain natriuretic peptide (BNP), a biomarker for cardiomyopathy and cardiac dysfunction, and increased heart size was reported in this mouse model^28^. We re-examined the expression of BNP as well as of other cardiac hypertrophy markers (ANP and β-MHC) in PA mice and confirmed that the three are upregulated (2–3 fold increase) in heart samples from 5 months-old mice (Supplementary Fig. 1). Neurological evaluation of the *Pcca* ^−/−^(A138T) mice has not been described. Preliminary behavior and locomotive studies performed in our lab indicate the existence of a mild age-dependent deficit in locomotion and motor coordination (unpublished observations).

**Figure 2:**
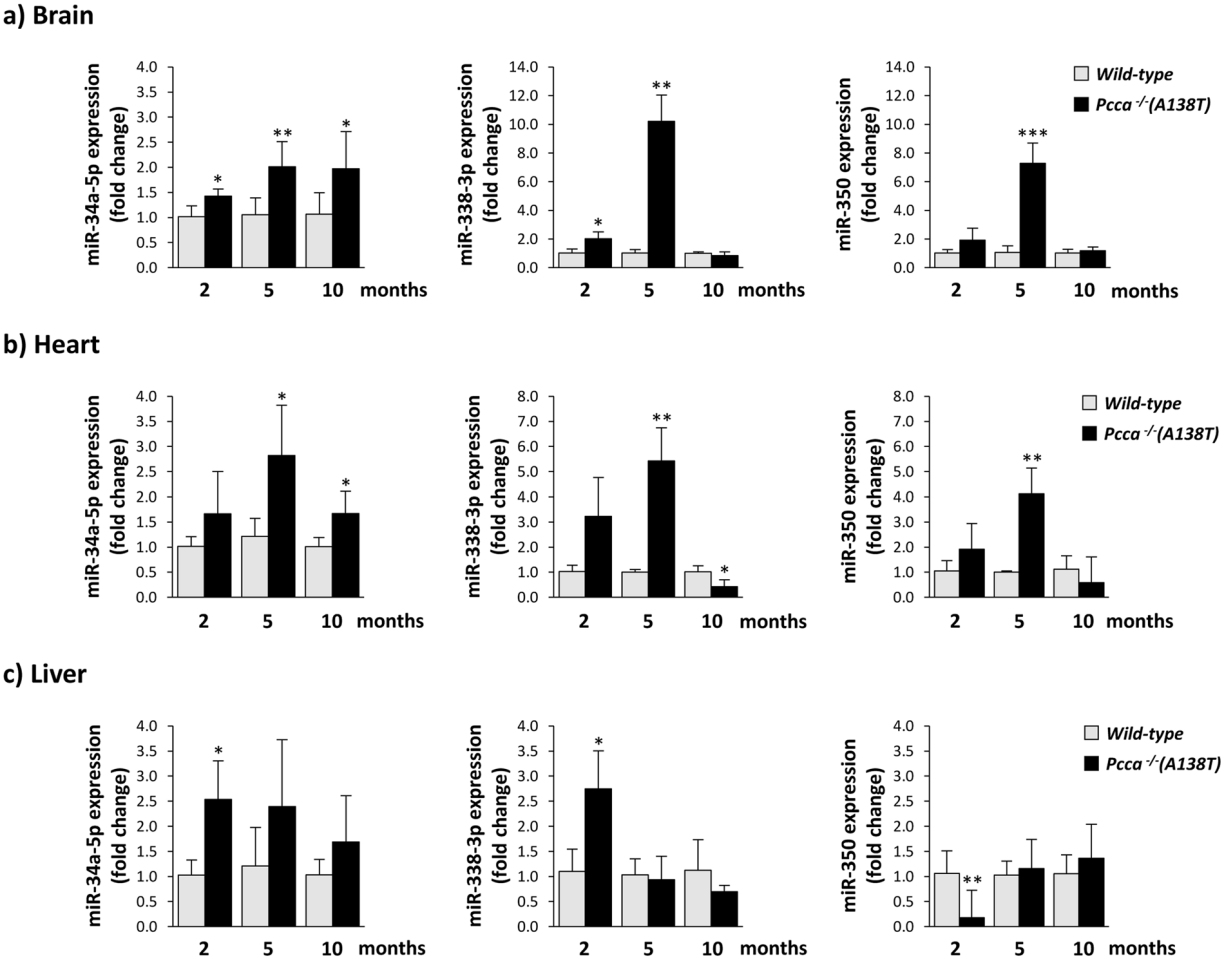
Relative expression levels of miR-34a-5p, miR-338-3p and miR-350 in brain (**a**), heart (**b**) and liver (**c**) tissues from PA mice at different ages. miRNA analysis was performed for wt and PA mouse samples (n = 4-5 per group) by qRT-PCR analysis. *p < 0.05, **p < 0.01, ***p < *0.001*.

To gain insight into the possible role of selected miRNAs in the organ pathophysiology of the disease we quantified the relative expression of miR-34a-5p, miR-338-3p and miR-350 in brain, heart and liver of PA mice and age-matched controls (two months-old, n = 4; five and 10 months-old, n = 5). The results confirmed that miR-34a-5p, miR-338-3p and miR-350 are upregulated in brain and heart tissues of PA mice at 2 and 5 months of age, while in 10 months-old mice miRNA levels tend to normalize (Fig. 2). In liver, we could detect modest variations in miRNA levels.

### Target genes are inversely down-regulated in PA mouse tissues

**Figure 3:**
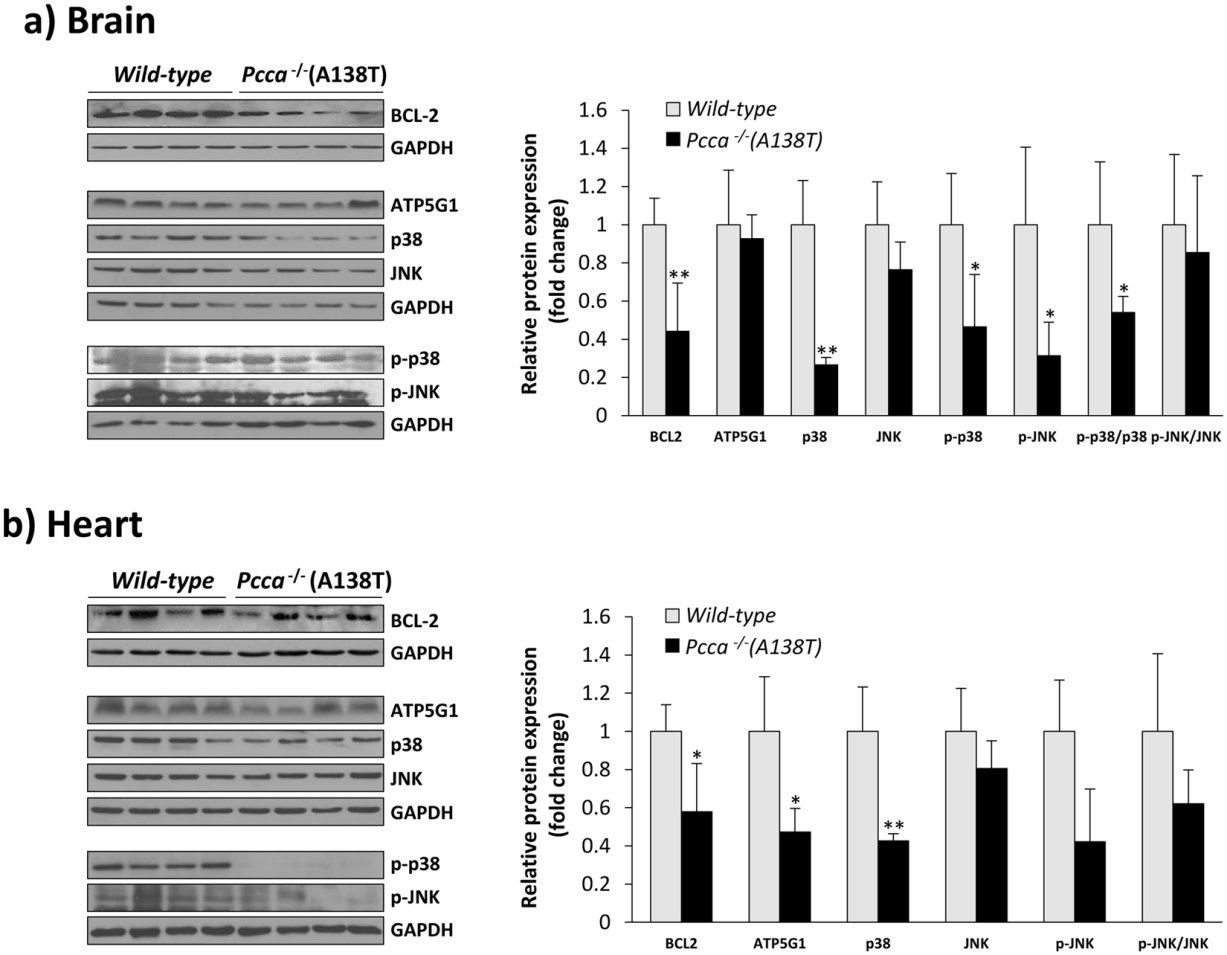
Expression levels of target genes by western blot analysis of the corresponding proteins in wt and PA brain (**a**) and heart (**b**) tissues. Representative cropped blots of BCL2 (miR-34a-5p target), ATP5G1 (miR-338-3p target), p38, JNK (miR-350 targets) and activated p-JNK and p-p38, along with the results of protein quantification performed by laser densitometry (n = 4–5 per group, 5 months-old mice). In each blot, GAPDH was used as loading control. Data represent mean ± standard deviation of three independent experiments. *p < 0.05, **p < *0.01*.

With the aim of identifying altered cellular pathways in PA that could contribute to the pathophysiology of the disease, thus identifying potential novel therapeutic targets, we analyzed the expression of target genes of the dysregulated miRNAs in heart and brain tissues of 5 months-old mice. These samples exhibit highest expression levels of the candidate miRNAs and represent relevant tissues in PA pathology. We selected genes involved in apoptosis, oxidative stress response and/or mitochondrial function, all of them experimentally validated targets of the corresponding miRNA as annotated in miRTarbase (http://mirtarbase.mbc.nctu.edu.tw/). We analyzed the expression of the following genes by western blot analysis of the encoded proteins: BCL2, NOTCH1 and SIRT1 (miR-34a-5p targets), ATP5G1 (miR-338-3p target) and JNK and p38 (miR-350 targets). We could not observe any significant differences in NOTCH1 or SIRT1 expression (data not shown). For the remaining targets, the results showed a decrease in protein expression levels in PA mice compared to controls (Fig. 3).

p38 and JNK belong to the mitogen-activated protein kinase (MAPK) superfamily, which play an essential role in cellular signaling as response to stress. We investigated whether there were changes in their activation (phosphorylation) state in addition to the observed decrease in total protein levels. As shown in Fig. 3, no phosphorylated p38 (p-p38) was detectable in heart samples from PA mice, while in brain a significant decrease was observed. Phosphorylated JNK (p-JNK) was decreased in brain and heart, in the latter not reaching statistical significance.

### Functional analysis in cellular models

**Figure 4:**
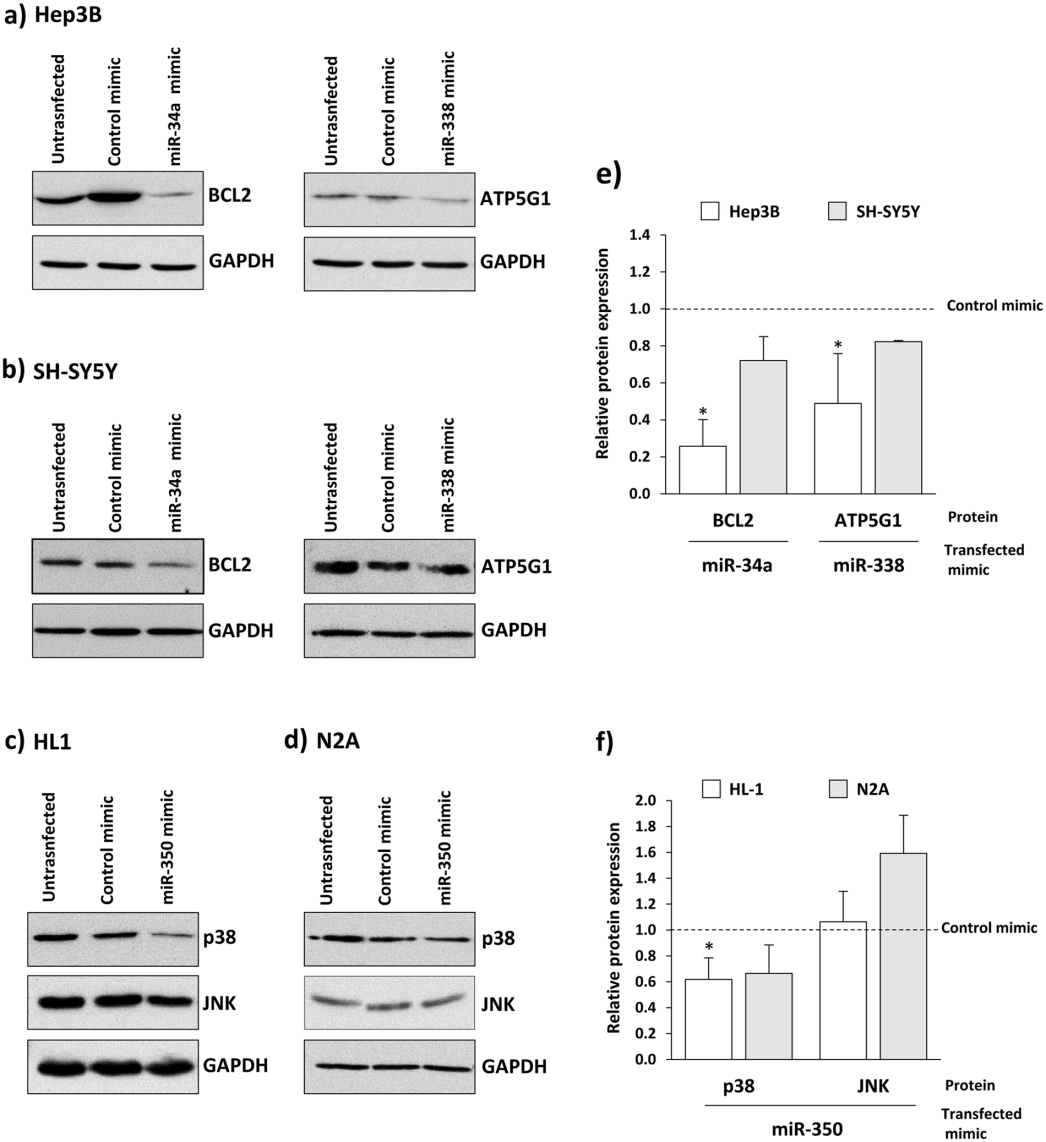
Western blot analysis of target genes expression after transfection with miRNA mimics. miR-34a-5p and mir-338-3p mimics were transfected in Hep3B (**a**) and SH-SY5Y cells (**b**) and miR-350 mimics in Hl-1 (**c**) and N2A cells (**d**). The figure shows representative cropped western blots of corresponding targets along with the results of protein quantification performed by laser densitometry (**e** and **f**). In each blot, GAPDH was used as loading control. Data represent mean ± standard deviation of three independent experiments. *p < *0.05*.

As previously mentioned, the regulation of the selected target genes by miR-34a-5p, miR-338-3p and miR-350 has been validated in experimental models, as referenced in miRTarbase^31–36^. To provide additional evidence, we performed overexpression and inhibition analysis *in vitro* using miRNA mimics and inhibitors. qRT-PCR studies in normal and patients’ fibroblasts indicated that none of the three candidate miRNAs are expressed in this cell type. Thus, functional analysis was carried out in Hep3B and SH-SY5Y cells (for miR-34a-5p and miR-338-3p), or in HL-1 and N2A cells (for miR-350, a rodent specific miRNA), cell lines in which the miRNAs are readily detected. After transfection, miRNA levels were assessed by qRT-PCR and targets were analyzed by western blot analysis. In each case, transfection of miRNA mimics or inhibitors resulted in a detectable increase or decrease, respectively, in the corresponding miRNA levels. Western blotting revealed the inverse downregulation of BCL2 with miR-34a-5p mimics and of ATP5G1 with miR-338-3p mimics. In HL-1 and N2A cells, p38 was also down-regulated after transfection with miR-350 mimics (Fig. 4). In these cellular systems, we could not detect significant changes in BCL2, ATP5G1, p38 or JNK protein levels after transfection with the corresponding inhibitors/antagomiRs (data not shown).

### Plasma miRNA alterations in PA patients

To translate the results obtained in PA mice to a clinical setting we sought to analyze the selected miRNAs in PA patients’ samples. As mentioned above, none of the three miRNAs are expressed in fibroblast samples. We decided to analyze their presence as circulating miRNAs in plasma samples, which would be relevant to investigate their potential as minimally invasive biomarkers. In PA mice, we could indeed detect the three miRNAs in plasma, at reduced levels compared to wt mice samples (Supplementary Fig. 2).

In PA patients, as a first step, we performed whole miRNome profiling analyzing the differences in miRNA levels between pooled plasma samples from patients at diagnosis (<1 month of age) and age-matched individuals (two pools of patients, and two pools of control individuals, each pool including 4–5 individual samples). miRNome profiling was performed using PCR panels with pre-aliquoted primers for 752 human miRNAs. A total of 352 miRNAs were quantifiable. Interestingly, more than half (60%) are not included in commercial kits for serum/plasma samples focused in abundant plasma miRNAs detected in adult individuals. A total of 73 miRNAs were found dysregulated (fold-change > or <1.5 in PA samples versus controls), 33 upregulated and 40 downregulated (Supplementary Table 1). Of note, miR-34a-5p and miR-338-3p were included among these miRNAs, as well as others (miR-29a-5p, miR-31-3p, miR-326 and miR-335-3p) also found dysregulated in liver samples of the PA mouse model (Table 1).

**Figure 5:**
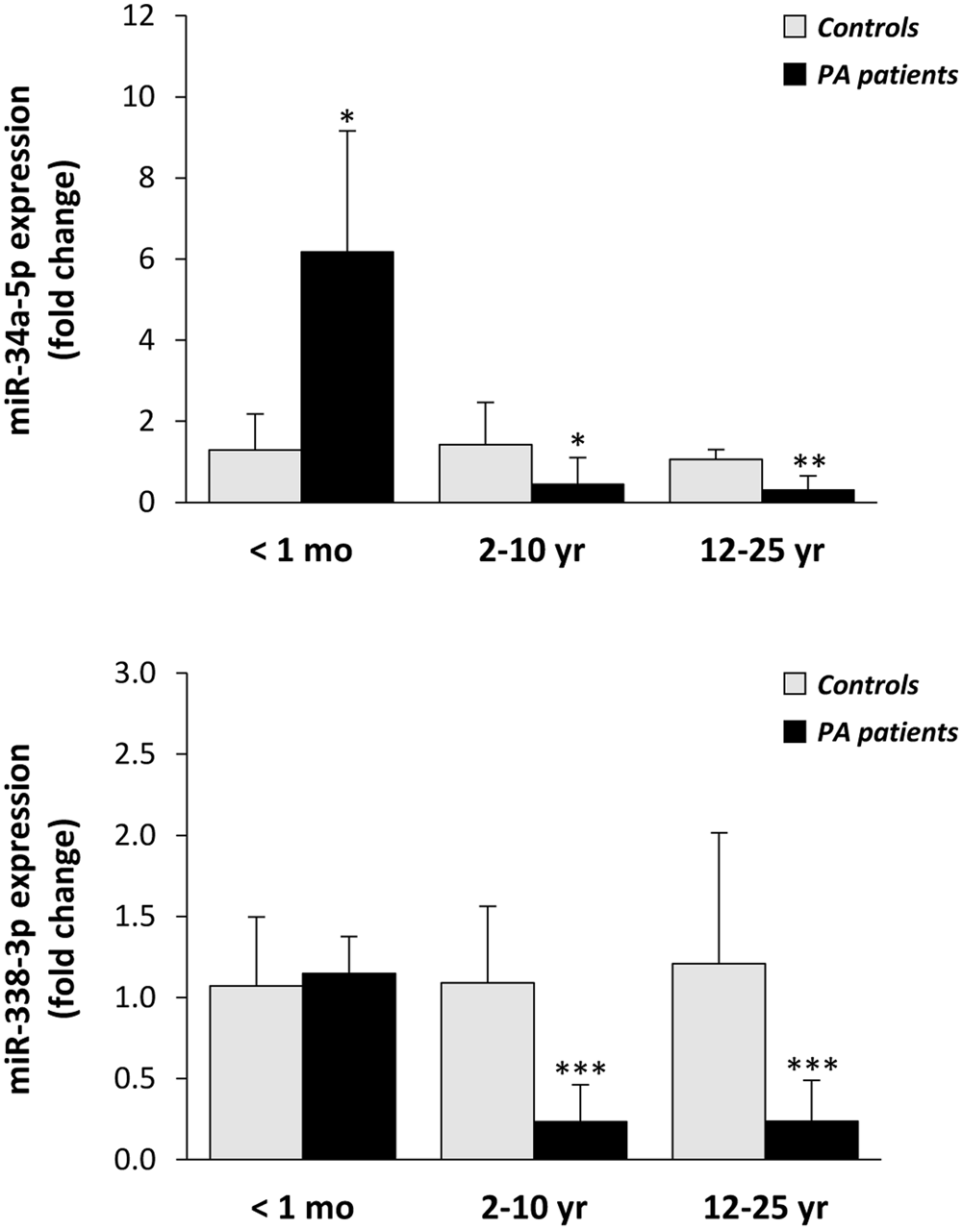
Relative levels of miR-34a-5p and miR-338-3p in plasma samples from PA patients. miRNA analysis was performed by qRT-PCR analysis. PA patient samples and matched controls were grouped according to age: <1 month-old (n = 8 per group), 2–10 year-old (n = 12 per group) and 12–25 year-old (n = 8 per group). *p < 0.05, **p < 0.01, ***p < *0.001*.

At a second stage, we examined the relative expression levels of miR-34a-5p and miR-338-3p in individual plasma samples in three different patient cohorts and their matched controls: 1) neonatal patients at diagnosis (<1 month old) (n = 8 per group); 2) 2–10 year-old patients at follow-up (n = 12 per group) and 3) 12–25 year-old patients at follow-up (n = 8 per group). Initial analysis of the results with this limited number of samples showed no significant differences in miRNA levels between *PCCA* and *PCCB* deficient patients or between female and male individuals, indicating that these are not main factors contributing to the altered miRNA levels reported. Taken together, the results show a significant increase in miR-34a-5p plasma levels in neonatal PA samples and a decrease of both miR-34a-5p and miR-338-3p in older patients (Fig. 5).

## Discussion

PA is a potentially lethal inborn metabolic disorder for which there is no efficient treatment. Although the genetic cause and the origin of the biochemical alterations are well known, the molecular pathogenesis of the disease resulting in multiorgan complications, the prognosis and the response to therapy are yet incompletely understood. Here, we describe the analyses of miRNAs and target genes in different tissues of the *Pcca* ^−/−^(A138T) mouse model of the disease, as well as the circulating miRNome in PA patients’ samples. The results identify miRNAs dysregulated in PA disease, some identified both in mouse and in human samples, and show target genes potentially involved in secondary pathologies downstream of the primary gene defect that could aid in the identification of therapeutic targets.

The correlation between PCC deficiency and altered miRNA expression is a priori elusive and could be related to the metabolic disturbances originated, which could act as signals for the modulation of miRNA levels. To date, knowledge of the underlying reasons and mechanisms behind changes in miRNA expression is still limited, although both transcriptional and post-transcriptional regulation of miRNA expression, as well as the effects of endogenous and exogenous compounds have been considered^37^. The age and tissue-specific differences in miRNA expression in PA mice suggest that different factors controlling the molecular adaptations to PA deficiency are at play, contributing to the complexity of the phenotype and presumably mediating the chronic pathological changes associated with the natural history of the disease.

Among the predicted targets of dysregulated miRNAs identified in PA mouse liver we focused on those involved in mitochondrial function, apoptosis, neurodegeneration or cardiomyopathy, alterations which are consistent with observed abnormalities in the mouse model and in patients^25, 26, 28^. Although the targets had been previously validated in different experimental systems, the results obtained in cell lines after transfection with miRNA mimics provide additional evidence of the specific miRNA mediated regulation. In our cellular assays, we could not observe a detectable effect using miRNA inhibitors. This might be due to the specific cell-type analyzed or to the lack of precise disease settings, or could be due to the presence of functionally redundant miRNAs compensating for the decreased miRNA. In this sense, it was recently shown that in the settings of heart disease where miR-34a is elevated, targeting the entire miR-34 family (that includes miR-34a, -34b and 34-c), proved more effective than targeting miR-34a alone^38^. Another factor to take into account are the rates of protein/mRNA turnover or of transcription/translation which could limit the detection by standard western blot analysis of modest protein decreases, which is what is expected for miRNA regulation^39^. However, the overall results are indicative of a regulatory effect of miR-34a-5p on BCL2, miR-338-3p on ATP5G1 and miR-350 on p38 in PA mice tissues.

The analysis of target genes allows the identification of signaling pathways that may aid in guiding biological therapeutic discovery. In this sense, our study represents a first step in unraveling novel miRNA regulated pathways and processes involved in the pathogenesis of PA disease. miR-338 is encoded intronically within the apoptosis-associated tyrosine kinase (AATK) gene in mice and humans. Both AATK and miR-338 are highly conserved genes, mainly expressed in the central nervous system, where they play a role during differentiation, apoptosis and possibly neuronal degeneration^40^. miR-338-3p has been found associated with axonal mitochondria where it regulates the expression of nuclear encoded mitochondrial mRNAs encoding subunits of the oxidative phosphorylation (OXPHOS) machinery^31, 41^. Recently, we described altered expression of OXPHOS complexes in the PA mouse^25^. The analysis of miR-338-3p expression in different parts of the brain and in cultured neurons of the PA mouse model and the correlation with levels of APT5G1 and other components of the OXPHOS system are subject to future experimental examination. In addition, miR-338-3p modulates the expression of axon guidance genes^42^, which should be evaluated in relation to PA pathology.

miR-34a-5p is described in many studies pointing towards a role in cancer proliferation, and it is also found dysregulated in muscular dystrophies, neurodegenerative diseases and myocardial dysfunction^43, 44^. miR-34a is known to regulate more than 30 oncogenes and, recently, a liposome encapsulated mimic of miR-34 (MRX34) has entered clinical trials, where it has demonstrated clinical proof of concept for solid tumors and hematological malignancies (clinicaltrials.gov). miR-34a is encoded in an intergenic region of chromosome 4 in mouse and in chromosome 1 in humans and is expressed ubiquitously (Human miRNA tissue Atlas: https://ccb-web.cs.uni-saarland.de/tissueatlas/). Among its well characterized targets is the antiapoptotic *Bcl2* gene^30, 45^, which was found inversely downregulated in PA mouse tissues. Other validated targets such as SIRT1 or NOTCH1 did not show differential expression, indicating that the regulatory effects exerted by miRNAs depends on the tissue and disease settings.

Contrary to miR-34a, miR-350 has been sparsely studied. It is an intronic rodent miRNA, encoded within the CEP-170 gene and not conserved in humans. miR-350 was found to induce heart hypertrophy in rat by repressing p38 and JNK stress kinases which leads to an increase in unphosphorylated NFATc3 localized in the nucleus, where it activates the expression of pathological hypertrophy markers^32^. In our study, we found a reduction in total and phosphorylated active forms of p38 and JNK both in heart and in brain of PA mice. Obviously, other factors are involved in the regulation of the phosphorylation of these stress kinases, but it is tempting to speculate that in PA mouse heart the downregulation of p38 and JNK by miR-350 may be responsible, at least in part, for the development of myocardial hypertrophy via NFAT signaling pathways. In humans, other miRNAs involved in NFAT regulation such as miR-133a-3p^46^, which was found differentially expressed in human PA plasma samples (Supplementary Table 1), are possible candidates to play a role in the development of cardiac hypertrophy.

The detection of miRNAs in plasma reveals their potential as signaling molecules and as disease biomarkers in PA. The pattern of expression (up or downregulation) in PA mouse plasma is not reciprocated in the different tissues, similar to what has been described in other diseases^47^. This indicates that circulating miRNAs may reflect a specific biological response rather than being a result of passive “leakage” from damaged cells.

Most relevant is the discovery in PA patients’ plasma of a specific miRNA signature that includes several miRNAs which were also found dysregulated in the mouse model of the disease, among them miR-34a-5p and miR-338-3p. Although the number of samples in this initial study is limited (due to the low disease prevalence) our results clearly show that these two miRNAs are present at significantly different levels in PA patients’ plasma compared to control individuals, in the three patient cohorts examined, at different ages and situations (at diagnosis or during follow-up as children or adults under standard dietary treatment). Even though there are tissue or species specific expression patterns of miRNA in the PA disease state, the coincidence of some dysregulated miRNAs in the PA mouse and in clinical samples underscore their relevance in PA. The PA patients’ plasma miRNome profiling study provides a focused subset of circulating miRNAs that may be further analyzed in larger patient cohorts at different ages and metabolic situations to evaluate their biomarker potential and their role in disease progression. Interestingly, plasma miR-34a-5p and miR-338-3p show a different profile in neonatal samples at diagnosis and in follow-up samples in older patients (Fig. 5). miRNAs are quite stable in serum and plasma and can be assessed easily using standard and affordable techniques, thus facilitating the observation of the evolution of the pathology at different stages of the disease and of the response to therapy^48, 49^.

This is the first analysis of the miRNA profile associated with PA. A recent study in the related disease methylmalonic aciduria (MMA), which is caused by a genetic defect in a subsequent step in the same metabolic pathway, identified miR-9-1 as downregulated in patients’ plasma, returning to normal levels after B_12_ treatment^50^. In a subsequent study miR-9-1 was shown to regulate proapoptotic BCL211 in neurons exposed to methylmalonate^51^. From the 26 miRNAs described showing differential expression in MMA plasma^50^, none overlapped those identified in this study in PA plasma. It may well be that there are distinct mechanisms for pathogenesis even in related diseases with similar biochemical and clinical alterations, although more in depth studies are needed to corroborate this.

In summary, our study provides evidence showing that miRNAs may modulate PA pathology through post-transcriptional regulation of genes involved in apoptosis, mitochondrial function and stress response. The results open new avenues to develop miRNA-based treatments. There is a pressing need for research into the sequence of events leading to progressive multiorgan complications, notably cardiomyopathy and neurological alterations. Elucidating the mechanisms that underlie these processes might result in therapeutic improvements, thereby improving the quality of life and length of survival of PA patients. In addition, as the role of specific miRNAs is established, and given their stable presence in plasma, miRNAs could potentially serve as complementary biomarkers along with biochemical parameters to guide follow-up and allow accurate clinical phenotype assessment in PA and related organic acidemias.

## Methods

### Mice handling

All mice used, wt and hypormorphic *Pcca* ^−/−^ *(*A138T), were adult males (2–10 months old) in an FVB background^28^. Mice were maintained on standard chow. Animal experiments were carried out in a pathogen-free environment at the Animal Facility of Centro de Biología Molecular Severo Ochoa, in accordance with the Spanish Law on Animal Protection. All animal studies were approved by the Institutional Animal Experimentation Ethical Committee (Universidad Autónoma de Madrid, reference CEI 963-A026) and by the Regional Environment Department (Comunidad de Madrid; reference PROEX 22/14). Genotyping was performed using genomic DNA isolated from tail biopsies as previously described^28, 52^.

### Human samples

Anonymized human plasma samples from PA patients, remnants of samples referred to the laboratory for diagnosis or follow-up, were used, with the corresponding informed consent. Overall, 19 samples were from *PCCB* deficient patients, 5 were *PCCA* deficient and 4 were from non-genotyped patients. Control plasma samples were obtained from Sera Lab Ltd (http://www.seralab.co.uk/), collected from consented anonymous donors. The study was approved by the Institutional Ethical Committee (Universidad Autónoma de Madrid). All human experimental methods were performed in accordance with the relevant guidelines and regulations.

### Cell culture and transfections

Hep3B, N2A and SH-SY5Y cells were cultivated according to standard procedures. Briefly, cells were maintained in Minimum Essential Medium supplemented with 1% glutamine, 5–10% foetal bovine serum (FBS) and antibiotics. HL-1 cardiomyocytes were cultured in Claycomb medium with 10% FBS supplemented with antibiotics and glutamine. 0.3 M ascorbic acid, 1 mM retinoic acid, 5 mg/ml insulin and 0.1 M norepinephrine were added to the medium. Cardiomyocytes cell plates had to be treated with 0.1% gelatin and 25 µg of fibronectin 24 h before seeding. The day before transfection, cells were seeded into 6-well culture cell plates (~200,000 cells per well). miRNA mimics (Exiqon) and antagomiRs (Exiqon) or miRVana Inhibitors (Ambion) were transfected using Lipofectamine 2000, following the manufacturer’s recommendations. 72–96 h after transfection, cells were harvested for miRNA analysis by qRT-PCR and for protein analysis by western blot.

### Sample preparation and RNA extraction

Wt and hypomorphic PA mice were sacrificed by cervical dislocation and brain, heart and liver tissues were immediately excised, snap-frozen in liquid nitrogen and stored at −70 °C until processed. Frozen tissues were pulverized using screws cooled in liquid N_2_ to obtain powdered tissue. Cultured cells were harvested by trypsinization and lysed by vortexing in lysis buffer supplied with the RNA isolation kit. Total RNA was extracted from the different powdered organs using miRCURY^TM^ RNA Isolation Kit - Tissue or Cell (Exiqon, Vedbaek, Denmark) according to the manufacturer’s instructions. The yield of total RNA was assessed using NanoDrop ND-1000 spectrophotometer (NanoDrop Technologies Inc, Rockland, DE, USA).

RNA isolation from mouse and human plasma samples was performed using the miRCURY^TM^ RNA isolation kit–Biofluids (Exiqon, Vedbaek, Denmark) following the manufacturer’s instructions. 200 µl of plasma were used and RNA was eluted in 20 µl. Subsequently, cDNA was synthesized using 4 µl of the RNA eluate.

### miRNA profiling

Total RNA from wt and PA mouse livers (n = 4 per group) and from human plasma samples (controls, n = 9 and PA patients, n = 10) was reverse transcribed to cDNA with miRCURY LNA^TM^ Universal cDNA synthesis kit II (Exiqon, Vedbaek, Denmark). cDNA samples from human plasma were pooled in two controls and two patient groups prior to miRNA profiling. miRNA profiling was performed with microRNA Ready-to-Use PCR Mouse & Rat or Human panel I + II V3 (Exiqon, Vedbaek, Denmark) which contain pre-aliquoted PCR primer sets (Locked Nucleic Acids, LNA^TM^ PCR primer sets) in 384-well PCR plates for 752 mouse and rat or human microRNAs, respectively. Real-time PCR amplification was performed with ExiLENT SYBR® Green master mix (Exiqon, Vedbaek, Denmark) in an ABI 7900HT instrument (Applied Biosystems, CA, USA). qRT-PCR reactions and analysis were carried out at Genomics Core Facility, Parque Científico de Madrid, Spain. The ABI software was used to obtain raw threshold cycle (Ct) value for each miRNA. Ct values ≥ 37 were considered negative. The Ct data were analyzed with the StatMiner 4.2.8 software (Integromics; Madrid, Spain). Relative quantification (RQ) or fold-change of each miRNA was calculated using the 2^−ΔΔCt^ method, with U6 snRNA, RNU1A, RNU5G, miR-103-3p, miR-191-5p, miR-423-3p and Let-7c-5p as internal controls for mouse liver analysis and with miR-let7a, miR-103a-3p, miR-15-5p and miR-191-5p for human plasma samples.

### Individual miRNA quantification

The relative expression of selected miRNAs in the different mice tissues, in mice or human plasma samples and in cultured cells was determined by qRT-PCR using the miRCURY LNA Universal RT microRNA PCR (Exiqon, Vedbaek, Denmark). Following reverse transcription, the cDNA template was amplified using microRNA-specific LNA primers for each miRNA and ExiLENT SYBR® Green master mix (Exiqon, Vedbaek, Denmark) in a LightCycler 480 instrument (Roche Applied Biosciences, In, USA), according to the manufacturer’s instructions. U6 snRNA and miR-423-3p and miR-23a-3p were used for normalization. The relative miRNA expression was quantified using the comparative threshold method after determining the Ct values for the reference and target genes in each sample set according to the 2^−ΔΔCt^ method. All reactions were performed in triplicate.

### mRNA quantification

In the analysis of pro-hypertrophic markers, 250 ng of total RNA isolated from mouse heart samples was retrotranscribed using the High Capacity RNA-to-cDNA kit (Applied Biosystems, CA, USA). The *Nppa* (coding for ANP), *Nppb* (BNP) and *Myh7* (β-MHC) genes were amplified using specific primers (available on request) using the Perfecta SYBR Green FastMix kit (Quanta Biosciences, Beverly, MA, USA) in a LightCycler480 II (Roche Applied Biosciences, In, USA) instrument. GAPDH was used as endogenous control. All samples were run in triplicate, and mRNAs relative expression were calculated using the 2^−ΔΔCt^ method.

### Bioinformatics tools

The following miRNA databases and target prediction tools were used to obtain information on selected miRNAs and to identify potential target genes: miRBase (www.mirbase.org), TargetScan (www.targetscan.org), MiRTarBase (http://mirtarbase.mbc.nctu.edu.tw/), microRNA.org (http://www.microrna.org/), DIANA TOOLS-mirPAth (http://diana.imis.athena-innovation.gr/DianaTools/index.php?r=mirpath), Ingenuity Target Explorer (http://targetexplorer.ingenuity.com) and Ingenuity Pathway Analysis (IPA, Ingenuity Systems, Qiagen, Hilden, Germany). miR2Disease database (http://www.mir2disease.org/) was used to review the association of dysregulated candidate miRNAs with human diseases.

### Western blotting

Proteins from mouse tissues were isolated by disrupting powdered mice organs in lysis buffer (50 mM Tris-HCl pH 7.5, 100 mM NaCl, 1 mM DTT, 1% Triton X-100, 0.1% SDS, 0.4 mM EDTA) using TissueLyser (Qiagen, Hilden, Germany) (two rounds of 90 seconds at 20 Hz) and centrifuged 30 min at 4 °C. The supernatant fraction was collected and protein concentration was determined by the Bradford method (Bio-Rad Laboratories, Hercules, CA, USA).

For western blot analysis from transfected cells, these were lysed by freeze-thawing in lysis buffer with protease and phosphatase inhibitors. Equal amounts of lysed extracts (50–100 µg protein) were loaded on a 10% or 12% SDS-polyacrylamide gel. After electrophoresis, proteins were transferred to a nitrocellulose membrane (iBlot® Gel Transfer Stacks, Regular) in an iBlot® Gel transfer device (Invitrogen, Carlsbad, CA, USA). Immunodetection was carried out using commercially available antibodies against BCL2 (1:500, Cell Signaling Technology, Danvers, MA, USA), SIRT1 (1:1,000, Millipore, MA. USA), NOTCH1 (1:500, Cell Signaling Technology, Danvers, MA, USA), ATP5G1 (1:1,000, Abcam, Cambridge, UK), p38, JNK, Phospho-p38 and Phospho-JNK (Cell Signaling Technology, Danvers, MA, USA, used at 1:1,000). Secondary antibodies used were goat anti-rabbit or goat anti-mouse (1:5,000, Santa Cruz Biotechnology, Santa Cruz, CA, USA). For loading control, membranes were immunostained with GAPDH antibody (1:5,000, Abcam, Cambridge, UK). Antibody binding was detected by enhanced chemiluminescence (GE Healthcare, Buckinghamshire, UK). Protein quantification was performed using a calibrated densitometer GS-800 (Bio-Rad Laboratories, Hercules, CA, USA).

### Statistical analysis

Data were presented as mean ± SD. To analyze significant differences, the distribution of the two groups was compared using two-tailed unpaired T-test distribution. *p* values below 0.05 were considered statistically significant: *p < 0.05; **p < 0.01; ***p < *0.001*.

## Electronic supplementary material

Supplementary information
