# Supplementary material for: Dysregulated miRNAs and their pathogenic implications for the neurometabolic disease propionic acidemia
Source: Sci Rep. 2017 Jul 18;7:5727. doi: 10.1038/s41598-017-06420-8 (PMC5516006; doi:10.1038/s41598-017-06420-8)

Supplementary information

"Dysregulated miRNAs and their pathogenic implications for the neurometabolic disease propionic acidemia"

Ana Rivera-Barahona, Alejandro Fulgencio-Covián, Celia Pérez-Cerdá, Ricardo Ramos, Michael A. Barry, Magdalena Ugarte, Belén Pérez, Eva Richard, Lourdes R Desviat

Supplementary Figure 1. Relative levels of Nppa (ANP), Nppb (BNP) and Myh7 (B-MHC) in heart samples from PA mice. The analysis was performed for wt and PA mouse samples (n=4-5 per group, 5 months of age) by qRT-PCR analysis. **p<0.05; **p<0.01*

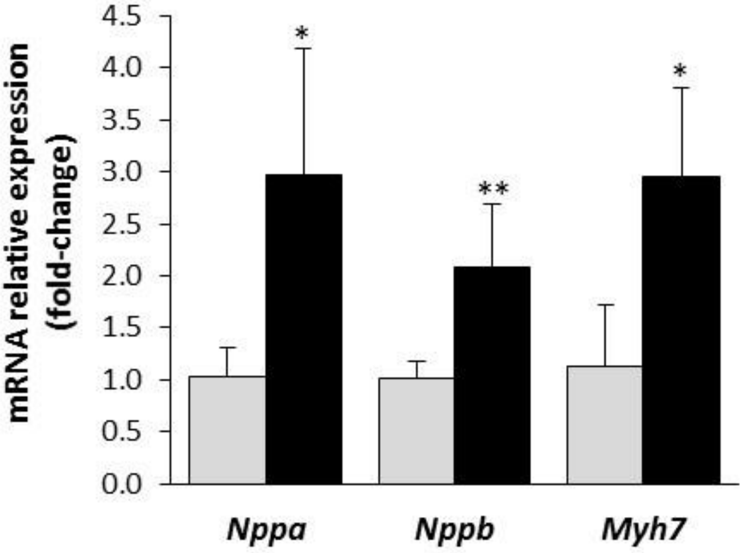

Supplementary Figure 2. Relative levels of miR-34a-5p, miR-338-3p and miR-350 in plasma samples from PA mice. miRNA analysis was performed for wt and PA mouse samples (n=4-5 per group) by qRT-PCR analysis. **p*<0.05*

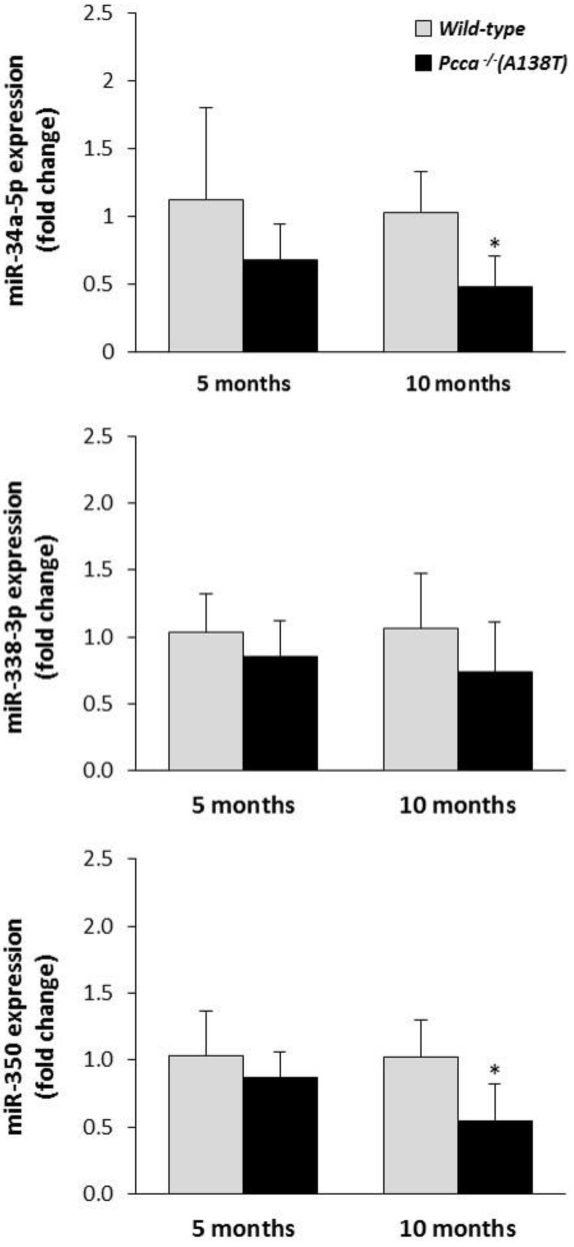

Supplementary Table 1. miRNAs detected in altered levels in PA patients' plasma samples

Detector	RQ	Tissue Expression*	Biological process	Disease	Validated targets
miR-519e-5p	51.7	Ubiquitous	Placental biomarker		
miR-382-3p	39.9	Ubiquitous		Rett syndrome, mild cognitive impairment	SOD2, DRD1
miR-31-5p	27.6	Thyroid, brain (cerebelum)	Apoptosis, proliferation, myogenesis	Cancer, DMD, Alzheimer's disease	DMD, MAP4K4, NFAT5
**miR-34a-5p**	9.6	Ubiquitous	Apoptosis, mitochondrial function	Cancer, cardiac hypertrophy, liver disease	BCL2, LDH, MAP2K1, NOTCH1, SIRT1, PPP1R10
miR-887-3p	6.2	Lymph nodes, liver		cancer	
miR-125b-5p	5.5	Brain, spinal cord	Apoptosis, proliferation	Cancer, Alzheimer's disease	BAK1, BCL2L2, MAPK1, TP53
miR-206	5	Muscle	Apoptosis, metabolism, proliferation	Cancer, myotonic dystrophy type I	G6PD, HSP60, NOTCH3
miR-143-3p	4.7	Oesophagus, bladder	Metabolism	Cancer	HK2
miR-96-3p	4.5	No data		Cancer, hypertrophic cardiomyopathy	
miR-132-3p	4.3	Brain	Apoptosis, neuronal function	Cancer, Alzheimer's disease, mild cognitive impairment	MAPK1, NOTCH1, SIRT1
miR-126-5p	4.3	Spleen, muscle, myocardium	Proliferation	Cancer, atherosclerosis	DLK1
miR-34a-3p	3.9	Ubiquitous			
miR-320d	3.9	Bone, brain (cerebelum)			
miR-10b-5p	3.9	Epididymus	Apoptosis, metabolism	Cancer	BCL2L11, PPARA
miR-548a-3p	3.7	Muscle, thyroid			
miR-320c	3.7	Ubiquitous			
miR-337-5p	3.4	Brain, bone, muscle			
miR-126-3p	3.4	Thyroid, myocardium, muscle	Apoptosis, metabolism	Cancer, cardiac disease	BCL2, SIRT1
**miR-31-3p**	3.3	Thyroid, brain		Cancer	
miR-152-3p	3.2	Thyroid, muscle		Cancer	DNMT1
miR-92b-3p	2.7	Ubiquitous		Cancer	
miR-323a-3p	2.7	Brain, spinal cord			
miR-548d-5p	2.6	Ubiquitous		Cancer, schizophrenia	
miR-543	2.5	Brain, muscle	Myogenesis	Cancer	TWIST1
miR-181d-5p	2.5	Ubiquitous	Apoptosis	Cancer	BCL2
miR-320b	2.4	Ubiquitous			
miR-151a-3p	2.4	Ubiquitous			
miR-30d-5p	2.4	Lungs, muscle, myocardium	Autophagy, apoptosis	Cancer, cardiac disease	BECN1, CASP3
miR-501-3p	2.1	Ubiquitous			
miR-505-3p	2.1	Spleen, thyroid, liver		Cancer	
miR-361-5p	2.1	Ubiquitous (brain, muscle)		Cancer	
miR-199a-3p	2.1	Thyroid, skin, bone	Metabolism	Cancer, cardiac disease	MTOR
miR-23b-3p	2	Epididymus		Cancer	
miR-29c-3p	0.7	Muscle, brain	Apoptosis	Cancer, Alzheimer's disease	BCL2, COL1A1, COL1A2
miR-598-3p	0.7	Brain, spinal cord			
miR-20b-5p	0.7	Thyroid, muscle	Proliferation, metabolism	Cancer	HIF1A, PPARG

miR-107	0.5	Brain, spinal cord, thyroid	Proliferation, lipid metabolism	Cancer, Alzheimer's disease, stroke	DICER1, HADHA, HIF1A, NOTCH2
miR-2110	0.6	No data			
miR-363-3p	0.6	Thyroid, brain, muscle	Apoptosis, cardiac function	Cancer	BCL2L11, CASP3, HAND1
miR-345-5p	0.6	Ubiquitous		Cancer	CDKN1A
miR-24-2-5p	0.6	No data			
miR-1224-3p	0.6	Spleen, liver			
miR-29a-3p	0.6	Muscle, brain	Apoptosis, fibrosis	Cancer, cardiac fibrosis, preeclampsia	BCL2, COL4A1, MCL1
miR-133a-3p	0.6	Muscle, myocardium	Proliferation, cardiomyocyte reprogramming, fibrosis	Cancer, cardiac hypertrophy	NFATC4
miR-128-3p	0.6	Brain, spinal cord, muscle	Apoptosis, proliferation	Cancer, Huntington disease, cognitive impairment	TGFBR1
miR-136-5p	0.5	Bone, brain, muscle	Apoptosis, metabolism	Cancer	BCL2, PPP2R2A
miR-548b-3p	0.5	Ubiquitous			
miR-33a-5p	0.5	Bone, muscle, brain	Proliferation, metabolism	Cancer	MYC, PPARA
miR-30e-3p	0.5	Muscle, myocardium	Survival	Cancer, heart failure	NFKBIA
miR-22-3p	0.5	Muscle, myocardium	Apoptosis, mitochondrial function	Cancer, cardiac disease, endometriosis	HDAC4, PPARA, SIRT1
miR-218-5p	0.4	Brain, spinal cord	Survival, proliferation	Cancer	RICTOR
miR-28-5p	0.4	Muscle, thyroid, myocardium	Apoptosis	Cancer	BAG1, NRF2
miR-376a-5p	0.4	Ubiquitous			
miR-378a-5p	0.4	Muscle, myocardium	Survival, proliferation	Cancer	NODAL
miR-200c-3p	0.4	Thyroid, skin	Proliferation, invasion	Cancer	ZEB1
miR-98-5p	0.3	Thyroid, muscle, brain			
**miR-326**	0.3	Ubiquitous	Apoptosis, proliferation, metabolism	Cancer, multiple sclerosis	BCL2L1, NOTCH1, NOTCH2, PKM
miR-96-5p	0.3	Epididymus, thyroid, skin	Apoptosis, metabolism	Cancer	FOXO1, FOXO3
**miR-335-3p**	0.3	Muscle, spleen		Cancer	
miR-99a-3p	0.2	Ubiquitous			
miR-455-5p	0.2	Bone, brain, spinal cord			
miR-766-3p	0.2	Ubiquitous		Cancer	
miR-337-3p	0.2	Spleen, muscle, liver		Cancer	
miR-331-3p	0.2	Muscle, thyroid, brain	Apoptosis, proliferation	Cancer	HOTAIR, NRP2
**miR-338-3p**	0.2	Brain, spinal cord	Apoptosis, mitochondrial function, neuronal growth	Cancer, ALS	COX4I1, HIF1A, MAP1A, PREX2A, ATP5G1
miR-95-3p	0.1	Muscle		Cancer	CELF2
miR-191-3p	0.1	Ubiquitous		Cancer, Alzheimer's disease, myocardial infarction	
miR-221-5p	0.1	Ubiquitous		Cancer, preeclampsia	
miR-500a-5p	0.06	Ubiquitous			
miR-493-3p	0.05	Ubiquitous		placental biomarker	
miR-138-5p	0.05	Brain, spinal cord		Cancer	
**miR-29a-5p**	0.03	Muscle, thyroid		Cancer, hypertrophic cardiomyopathy, preeclampsia	
miR-182-5p	0.02	ubiquitous		Cancer, heart failure	

miRNAs in bold were also differentially expressed in PA mouse liver

*** Human miRNA tissue Atlas: https://ccb-web.cs.uni-saarland.de/tissueatlas/**

Extended data Figure 3 (uncropped gels)

BRAIN

BCL2

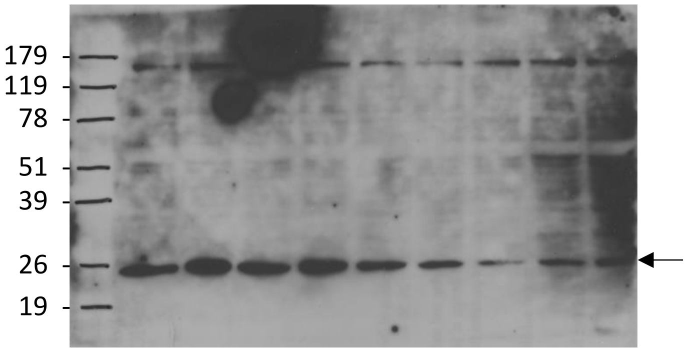

GAPDH

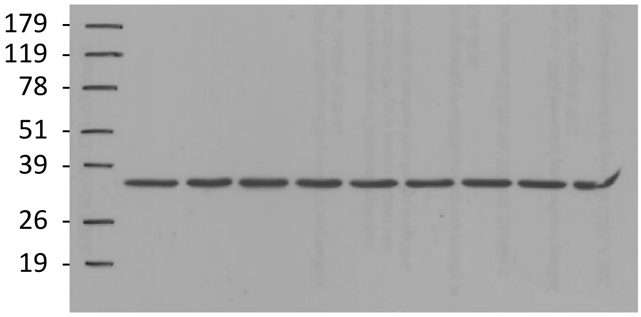

p38

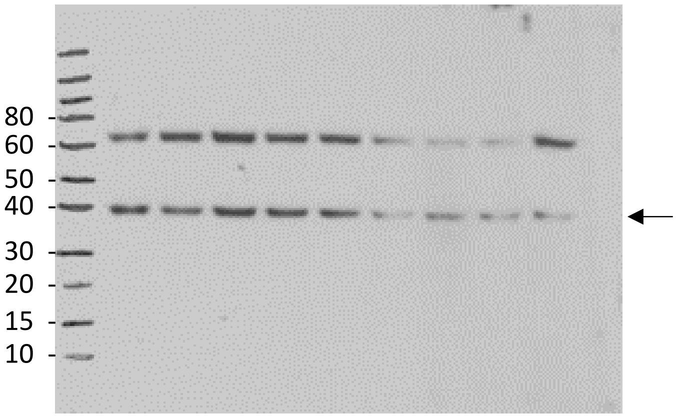

JNK

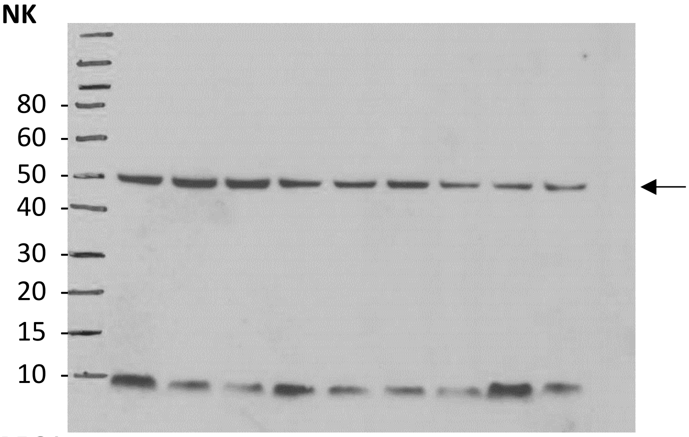

ATP5G1

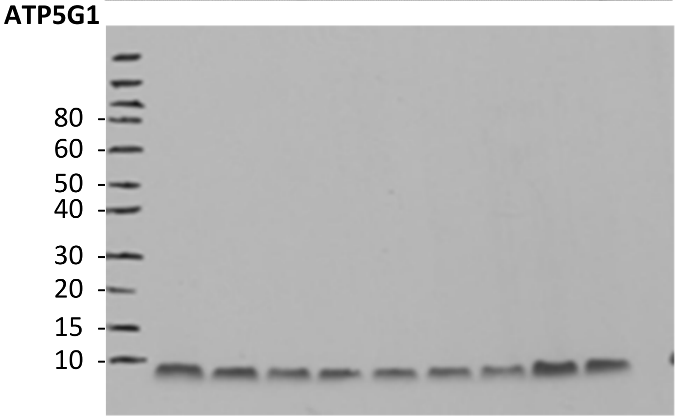

GAPDH

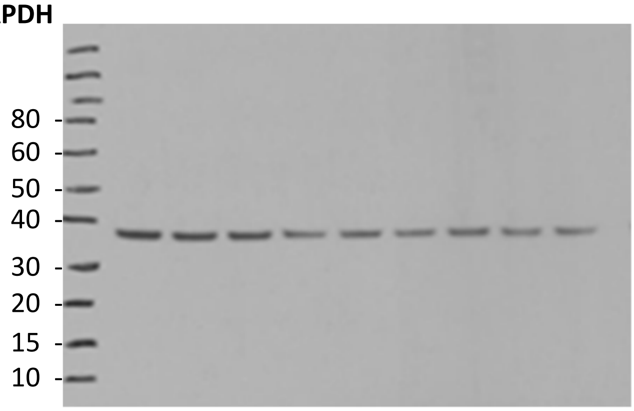

Extended data Figure 3 (uncropped gels)

HEART

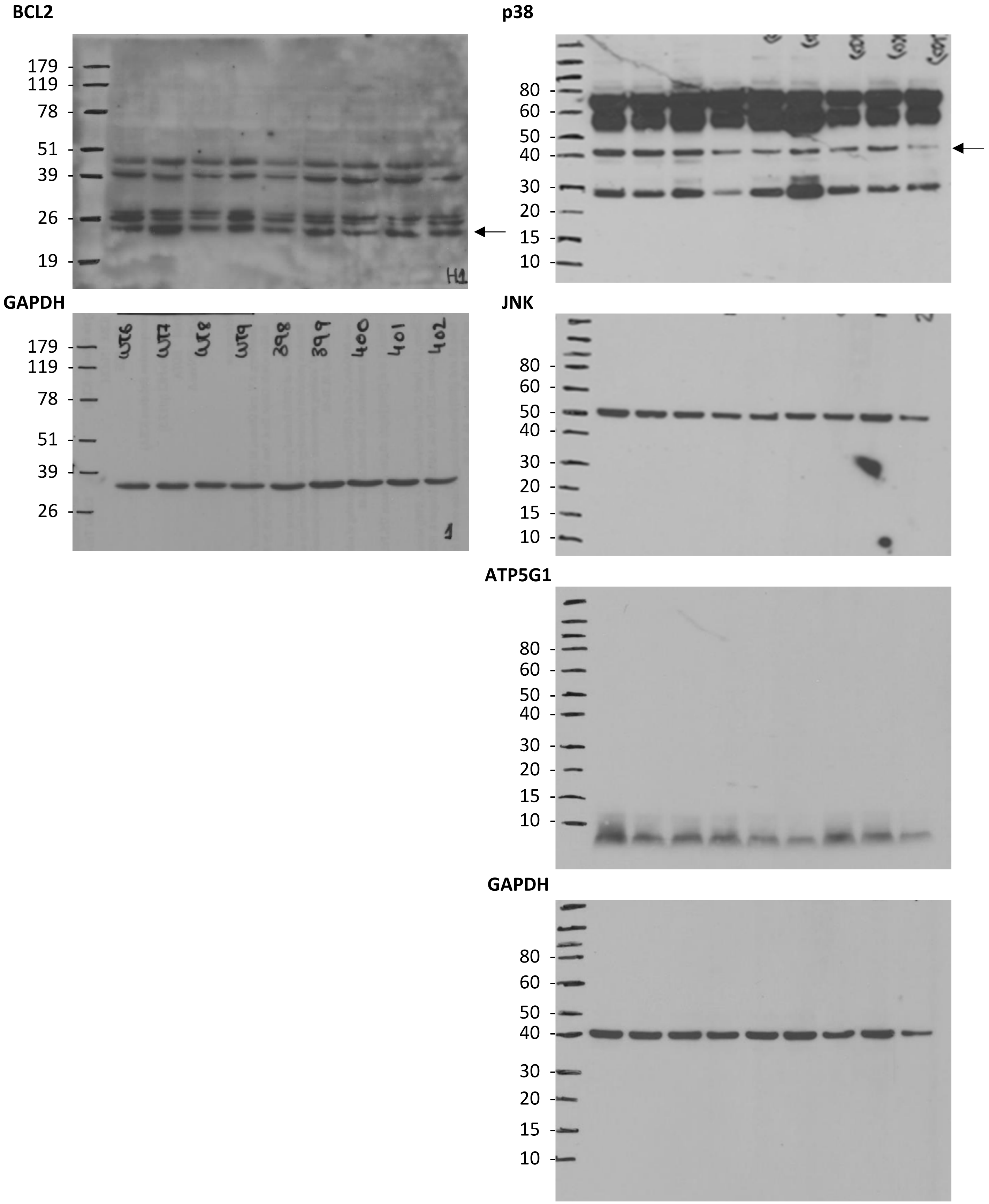

Extended data Figure 3 (uncropped gels)

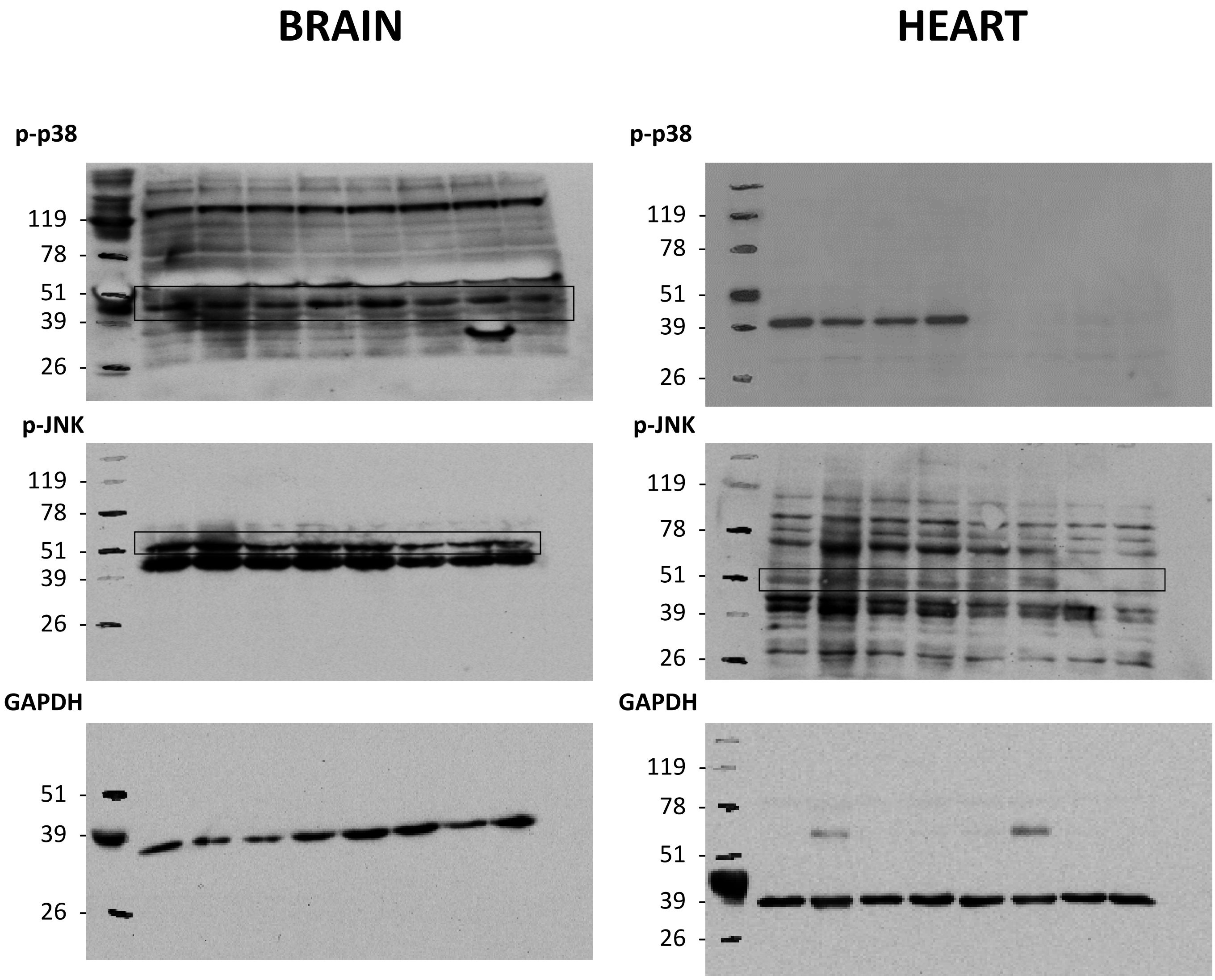

Extended data Figure 4 (uncropped gels)

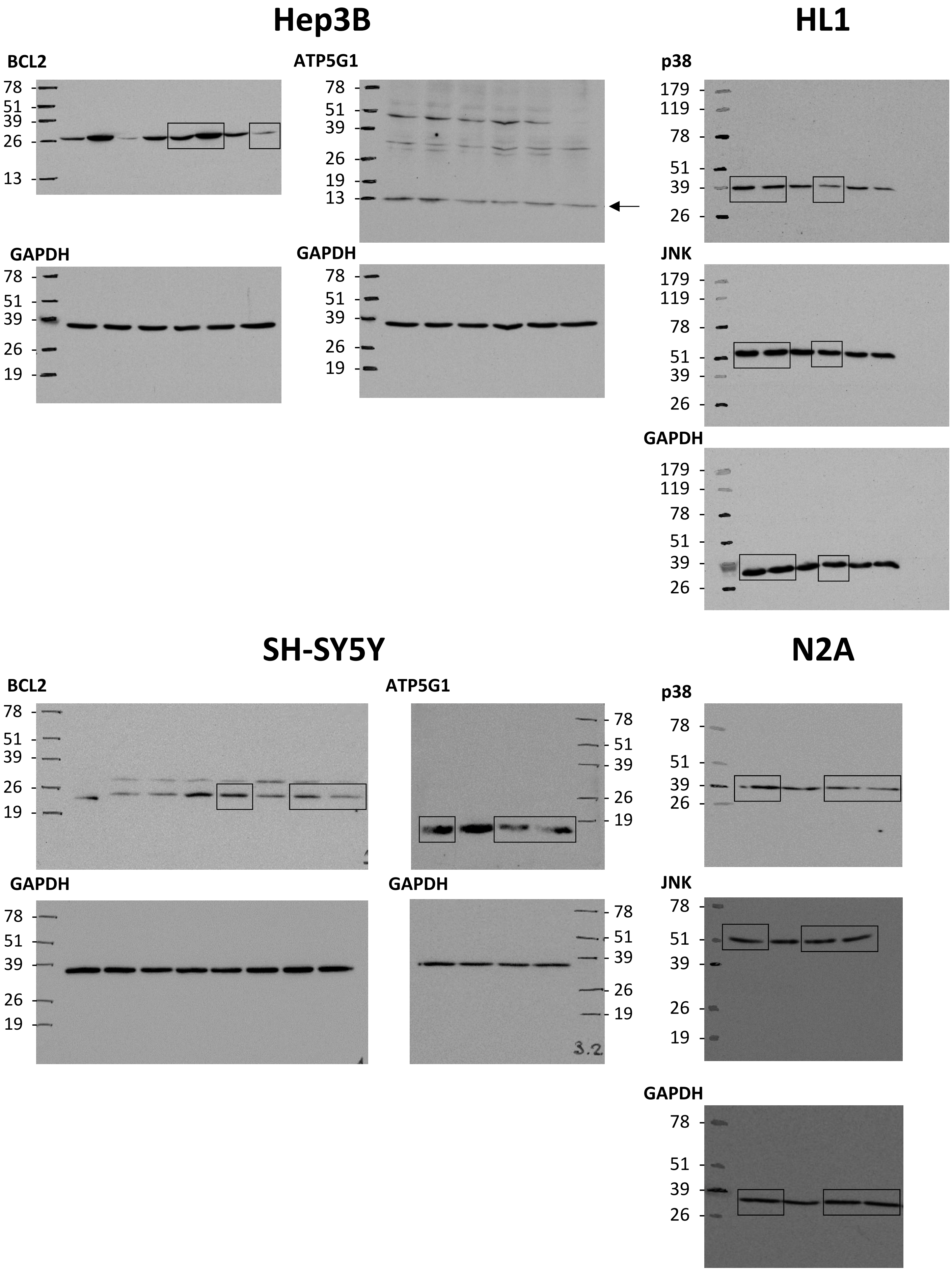

Supplement: Supplementary file 1 — Supplementary information [file 41598_2017_6420_MOESM1_ESM.pdf]
